# Strengthening Postabortion Family Planning Services in Ethiopia: Expanding Contraceptive Choice and Improving Access to Long-Acting Reversible Contraception

**DOI:** 10.9745/GHSP-D-15-00301

**Published:** 2016-08-11

**Authors:** Melaku Samuel, Tamara Fetters, Demeke Desta

**Affiliations:** a Ipas, Addis Ababa, Ethiopia; b Ipas, Chapel Hill, NC, USA

## Abstract

Where unmet need for the safest, most effective, and long-acting reversible contraceptives (LARCs) is very high, the health system and partners need to implement problem-solving, locally feasible, and comprehensive family planning delivery strategies. Because young and unmarried women are most at risk for unintended pregnancy and repeat abortion due to poor access to contraceptive services, postabortion family planning (PAFP) is a key component in such strategies. In Southern Nations, Nationalities, and People’s Region, Ethiopia, Ipas implemented health system strengthening efforts from fiscal year (FY) 2010 (July 2009 to June 2010) to FY 2014 (July 2013 to June 2014) to improve the quality of PAFP services and expand method choice in 101 public facilities. The intervention significantly improved PAFP uptake at the project sites. Specifically, the proportion of abortion clients receiving LARCs progressively improved during the intervention period. The proportion of abortion clients who left the facilities with a contraceptive method increased from 58% in FY 2010 to 83% in FY 2014. The share of method mix for LARCs rose from 2% in FY 2010 to 55% in FY 2014, while the share for condoms, injectables, and oral contraceptives declined from 98% to 45%. Implant use rose from 2% in FY 2010 to 43% in FY 2014, while the use of intrauterine devices increased from 0.1% in FY 2010 to 12% in FY 2014. A larger proportion of PAFP users received LARCs at health centers, where midwives and nurses are the primary providers, than at hospitals (59% versus 37%, respectively). A broader method mix can satisfy clients with a variety of needs, a key factor for higher uptake of more effective methods and program success. Further evidence-based interventions need to be implemented to improve the quality of PAFP in a feasible and replicable strategy that addresses unmet need for modern contraceptive methods.

## Abstract (precis)

In Ethiopia, a comprehensive strategy to improve postabortion family planning services has produced overall improvement in the uptake of postabortion family planning and a rise in the choice of more effective long-acting reversible contraceptives to produce a more balanced method mix.

## INTRODUCTION

In Ethiopia, as throughout the world, low levels of contraceptive use lead to high levels of unintended pregnancy, the root cause of induced abortion. The first nationally representative study in Ethiopia, conducted in 2008, revealed that 42% of all pregnancies were unintended, and an estimated 382,500 induced abortions were performed over a 1-year period, for an annual rate of 23 abortions per 1,000 women of reproductive age.^1^ Many of these abortions were still performed with questionable levels of safety and efficacy outside of health facilities. Women seeking induced abortion had a mean age of 23, and the majority (57%) were single. Among women seeking induced abortion, only 24% reported contraceptive use prior to the current pregnancy. The same study also found that 1 of every 4 facilities in the country did not have designated postabortion contraceptive services for women who had received abortion care.^1^

In 2008, among women seeking induced abortion, only 24% reported contraceptive use prior to the current pregnancy.

It is estimated that as many as 95% of unintended pregnancies in Ethiopia occur among women who do not practice contraception at all.^2^ The national Demographic and Health Survey in 2011 found that 1 of every 4 married women had an unmet need for family planning, and nearly 29% of most recent births and current pregnancies were reported as either mistimed or unwanted.^3^

The high unmet need for contraception coupled with high numbers of safe and unsafe abortions testifies to the need for stronger routine contraceptive services and highlights the potential benefits of strengthening postabortion contraceptive services. Postabortion family planning (PAFP) involves provision of voluntary contraceptive counseling and methods to women after abortion care, whether for induced abortion or the treatment of complications from an unsafe abortion, to reduce unintended pregnancies and repeat abortions. International organizations in the field of reproductive health and health researchers have posited that PAFP services that respect women’s sexual and reproductive rights are an ideal way to reach sexually active women with unplanned pregnancies, including young women who may have had limited exposure to modern contraception.^4^^–^^9^ Studies and interventions conducted in different parts of the world have shown that PAFP improves women’s knowledge of the benefits of contraception and increases uptake of contraceptive methods immediately after abortion, when return to fertility is almost immediate. Women’s motivation to continue using a method is also likely to increase after leaving the facility.^10^^–^^14^

Although many factors may constrain family planning programs, a limited method mix has been shown to adversely affect both contraceptive acceptance and continuation postabortion as well as the overall achievement of a national family planning program.^15^^,^^16^ Family planning programs, especially those for PAFP, should offer a range of methods in order to meet the various needs of their clients.^4^^,^^7^^,^^8^ Evidence has shown that introducing a wider range of contraceptive methods in PAFP significantly increases the proportion of clients leaving the facility with a method.^12^^,^^17^ The benefits of contraception can be particularly important for postabortion contraceptive acceptors: Many postabortion clients face high risk of maternal mortality due to high parity or the likelihood that their pregnancies could end in unsafe abortion.^18^ Moreover, comprehensive PAFP counseling is likely to promote the choice of more effective methods as women learn more about less common contraceptive options.^19^^–^^21^ Including more effective methods in the method mix can enhance program impact; use of long-term, highly effective methods such as intrauterine devices (IUDs) and contraceptive implants is likely to prevent more unplanned pregnancies and decrease demand for abortion.^20^^,^^22^

Cognizant of the broader benefits of PAFP services to the reproductive health of women and the Ethiopian National Family Planning Program, the international NGO Ipas has implemented an evidence-based health systems strengthening intervention to improve PAFP uptake. The intervention has focused on achieving a more balanced contraceptive method mix by increasing use of long-acting reversible contraceptives (LARCs). Although the intervention took place in 4 major regions of the country, this article presents only the approaches, achievements, and lessons learned from 101 public health facilities in the Southern Nations, Nationalities, and People’s Region (SNNPR) of Ethiopia between fiscal year (FY) 2010 (July 2009 to June 2010) and FY 2014 (July 2013 to June 2014).

## MOVING FROM POSTABORTION CARE TO COMPREHENSIVE ABORTION CARE

Since 2002 Ipas Ethiopia, as one of the major partners of the Ethiopian health sector, has been supporting the Ministry of Health (MOH) to improve the delivery and quality of abortion care and PAFP services. Before revision of law in 2005, all facilities were expected to provide only postabortion care (PAC), including postabortion contraception, for the management of incomplete abortion and complications resulting from induced or spontaneous abortion. Following legal reform that expanded the indications for safe abortion services,^23^ the MOH and its technical partners worked extensively to introduce a comprehensive abortion care (CAC) model. The model is an integrated set of sexual and reproductive health services that include safe induced abortion for all legal indications, treatment of incomplete and unsafe abortion, counseling, and provision of contraception and other reproductive health services as needed. Despite these efforts, however, service data revealed a low rate of uptake of contraception after abortion care and a highly skewed method mix in most facilities.

A baseline facility assessment in 2008 identified a number of gaps and barriers. Only 47% of health centers in SNNPR provided postabortion contraception—most often because they provided no abortion care at all.^24^ Only 8% of all facilities offered IUDs, and only 28% offered implants. Factors identified as limiting the availability or quality of PAFP services included:

- **Gaps in service providers’ knowledge:** Although PAC services were widespread in the country at the time, an assessment of these services conducted in 2000 found that more than three-quarters of PAC providers had never received any special training in family planning counseling or service delivery.^25^ A later assessment of facilities in SNNPR, conducted in 2008, reported that only half of health facilities had providers capable of providing LARCs or permanent methods.^24^
- **Shortages of contraceptive commodities, equipment, and supplies:** Keeping a wide range of contraceptive methods in stock in facilities and in the abortion services areas was another weakness in PAFP service. At the time of the baseline assessment, less than one-quarter of facilities had implant insertion and removal kits, and only 5% had insertion and removal kits for IUDs.^24^ Oral contraceptives were universally available, however, and injectable contraceptives were available in 97% of facilities.^25^
- **Lack of designated service delivery space for PAFP:** Most facilities (88%) provided PAFP services in the maternal and child health or family planning units, separate from the locations of PAC services.^25^ Most clients were asked to go elsewhere in the facility to receive contraception, were given a referral, or were asked to return at another time. Many health centers (and some hospitals) provided abortion care and PAFP services in delivery rooms, often with a limited mix of contraceptive methods, if they were available at all.
- **No special focus for young women and adolescents:** World Health Organization (WHO) guidance recommends that all reproductive health programs and services address the special needs of adolescents and young women.^4^ Young and adolescent women are more vulnerable to unwanted pregnancy and unsafe abortion because they are less likely to have initiated contraception. Therefore, services for these women need to be both more comprehensive and focused on their needs. However, site observations in the project area indicated that most facilities were unable to provide effective and accessible services to young clients that took into account their special needs, such as privacy and confidentiality throughout the continuum of their care. One major problem was a lack of training and continuing support to health care providers to improve their knowledge and skills on the package of interventions and service delivery approaches for young and adolescent women.
- **Poor referral linkages between facilities and community-based programs:** Well-established referral linkages and technical assistance agreements between abortion-providing facilities and community partners that provide contraceptive services and information were often lacking, contributing significantly to underutilization of services.^24^

## KEY COMPONENTS OF THE INTERVENTION

After identification of these major service delivery gaps, Ipas staff and MOH partners designed and implemented problem-focused interventions to improve service delivery standards and provide accessible, high-quality PAFP services acceptable to all clients. The ultimate goal of the intervention was to improve all components of comprehensive abortion care. However, increasing the uptake of postabortion contraception and improving the contraceptive method mix, with special attention to LARC methods, was a major focus.

Increasing uptake of postabortion contraception and improving the method mix, with special attention to LARCs, was a major focus of the intervention.

The effort to increase uptake of PAFP and of LARC methods especially included 8 key elements:

- Integration of CAC and comprehensive contraceptive trainings
- Improving the availability of contraceptive commodities and related equipment
- Renovation, refurbishment, and reorganization of CAC and PAFP service delivery sites
- A quality improvement initiative
- Promotion of youth-friendly services
- Clinical mentoring and programmatic supports to providers
- Strengthening community–facility linkage through involvement of health extension workers
- Integrating reproductive autonomy into contraceptive method selection

### Integration of Comprehensive Abortion Care and Comprehensive Contraceptive Trainings

Although many abortion care providers had received some exposure to postabortion contraceptive provision, the project made a renewed effort to improve their skills. All CAC providers participated in an updated training on comprehensive contraception. The new curriculum emphasized scientific and technical updates concerning LARC methods, as this had been identified as a particular weakness. Contraceptive trainings employed both didactic and practical training to improve the clinical skills necessary to provide IUDs and implants. The curriculum stressed the elements of high-quality family planning counseling services as recommended by WHO.^15^ These include a renewed emphasis on free and voluntary choice of a method. The contraceptive/LARC training was primarily designed for mid-level providers—midwives, nurses, and health officers—the cadres largely responsible for providing reproductive health services, including abortion care, at the primary health care level.

Between July 2009 and June 2014, 545 mid-level providers from 101 facilities received training in comprehensive contraception.^26^ Almost all of these providers were also trained to provide comprehensive abortion care. A pilot initiative designed to expand integrated PAC services at the primary care level through support to midwives (such as in-service training and supervisory and logistics support) led to improved access to PAFP services—70% of PAC clients received PAFP services.^27^ By comparison, before the initiative there was no routine system of postabortion counseling in the region and only minimal efforts to break the cycle of unintended pregnancy and unsafe abortion.

### Improving the Availability of Contraceptive Commodities and Related Medical Equipment

Oral contraceptives, injectable contraceptives, IUDs, contraceptive implants, and condoms should be available at all facilities; permanent methods performed surgically—tubal ligation and vasectomy—are available only at hospitals. Based on gaps identified in the intervention facilities, facilities began to regularly receive supplies of IUDs, implants, and essential family planning kits (such as medical equipment and supplies required for insertion and removal of IUDs and implants). In addition, hospitals received mini-laparotomy and no-scalpel vasectomy kits. From 2008 through 2012, a total of 92 IUD insertion and removal kits and 12 voluntary surgical contraceptive kits (mini-laparotomy and no-scalpel vasectomy kits) were distributed to the facilities in SNNPR.^26^ Ipas also provided the MOH regional health system with technical assistance to establish a sustainable supply of commodities and equipment for comprehensive abortion and contraceptive provision. Pharmacy personnel in health facilities received orientation to the products and to forecasting and procurement through the government supply system.

### Renovation, Refurbishment, and Reorganization of Service Delivery Sites

Service delivery experiences in various countries have shown that providing counseling and postabortion contraception at the same time and location where abortion services are provided can reduce missed opportunities and enhance acceptance of PAFP.^14^^,^^20^ With these benefits in mind, Ipas provided financial, material, and technical support to 53 facilities to renovate, refurbish, and reorganize service sites based on the identified needs in each facility. The work significantly improved privacy, confidentiality, infection prevention practices, and the organization of services to provide abortion and PAFP services. The upgraded service delivery areas also improved providers’ motivation and commitment to provide high-quality, integrated care.

### The Quality Improvement Initiative

This initiative was pilot-tested in 12 facilities (9 health centers and 3 hospitals) randomly selected from among Ipas-supported sites in SNNPR. During the pilot-test period, all progress and changes in the quality of abortion care indicators were monitored and documented at regular intervals. At the conclusion of the pilot program, significant improvement had been achieved in most of the key quality indicators for CAC services, including PAFP.^28^ One objective of the pilot-test was to produce recommendations for scaling up simple, practical quality improvement tools that can be integrated into the existing program monitoring system. Based on lessons learned from the pilot exercise, such practical monitoring tools as simple site-level indicators and on-site exercises for service improvement were integrated into the routine supervisory processes and replicated in the other sites in the region. Reminders were posted in CAC service areas on such quality-of-care points as abortion procedure protocol and clients’ rights to informed choice, privacy, and confidentiality during service delivery.

### Promotion of Youth-Friendly Services

Almost 60% of women receiving abortion care at Ipas-supported sites are under age 25. Thus, it was imperative to raise the awareness of service providers about the needs and circumstances of young women. To improve access to comprehensive abortion care for young women, a pilot project was initiated in 12 of the SNNPR project facilities. This initiative focused on:

1. Training providers in accord with national standards and guidelines
2. A minimum service delivery package of youth-friendly services
3. Support for reorganization of services for potential youth beneficiaries^29^

The facilities received technical and financial support to make the service delivery setup more convenient for young clients and to provide contraceptives in the abortion procedure rooms, which helps to ensure privacy and confidentiality during service delivery. Also, facilities were equipped with information materials, furniture, and audiovisual aids to attract and serve young people.

Facilities were encouraged to integrate comprehensive abortion care with all other reproductive health services, such as care for sexually transmitted infections, including HIV. Efforts were also made to establish and improve the referral networks between other facilities, community-level health services, and youth-focused organizations. After reviewing the lessons and challenges learned from the pilot initiative, all other facilities in the project sites integrated the service approach into their CAC services.

### Clinical Mentoring and Programmatic Supports to Providers

Training alone does not always ensure that providers are competent and confident and able to put all their skills into practice. In this intervention newly trained providers received needs-based on-site clinical mentoring by senior and seasoned providers from the same facility. The providers requiring clinical support were identified at the end of the training or while providing services and linked with on-site mentors. The mentors provided more intensive clinical support, as frequently as necessary. In addition, all newly trained providers were contacted within 3 to 4 weeks after training to ascertain whether they had begun offering services and to provide clinical and programmatic support. To promote sustainability, all programmatic support was coordinated with MOH regional staff and facility management.

### Strengthening Community–Facility Linkage Through Involvement of Health Extension Workers

In Ethiopia health extension workers (HEWs) have responsibility for community-level reproductive health services, including counseling and provision of some contraceptive methods and referrals for others. A study on utilization of PAC services found that community providers can play an important role in improving community awareness of abortion-related issues, overcoming stigma, and disseminating information on the availability of services.^30^ Ipas conducted orientation workshops for 368 HEWs on long-acting contraception, myths and misconceptions about IUDs, unwanted pregnancy, and comprehensive abortion care. The trainings focused not only on skill building but also on creating a strong referral linkage between health facilities for abortion care and community health programs for contraceptive services, particularly for clients seeking long-acting or permanent methods. To improve HEWs’ counseling skills, the WHO decision-making tool was distributed to all HEWs in the intervention areas. In addition, abortion care providers and reproductive health coordinators from the MOH participated in orientation workshops to develop formal agreements for providing mentoring for HEWs and establishing more effective referral linkages between facilities and community health services.

Trainings for HEWs focused not only on skill building but also on creating a strong referral linkage, particularly for clients seeking long-acting or permanent methods.

### Integrating Reproductive Autonomy into Contraceptive Method Selection

While this intervention focused on eliminating barriers that impede postabortion contraceptive acceptance and contribute to a skewed method mix, it was important to do so while respecting and emphasizing women’s reproductive autonomy. All training packages include sessions on reproductive rights and effective counseling steps, emphasizing the responsibility of service providers to assure that clients can make informed, voluntary choices concerning their abortion procedures and contraception. Training for reproductive health officers and on-site exercises, adapted from the *COPE for Comprehensive Abortion Care* manual, provided information and practice for providers about clients’ rights to information, choice, and safe services.^28^

## METHODS

### Data Collection Methods and Tools

Through routine monitoring, project staff regularly collected and reviewed service data from all intervention facilities (78 facilities in FY 2010 and FY 2011, 95 in FY 2012 and FY 2013, and 101 in FY 2014). From FY 2010 to FY 2012, data were collected quarterly; in subsequent years, semiannually. Zonal health department staff members, along with Ipas program coordinators, were responsible for data collection, extracting information from the abortion services logbook using a data collection form developed for this purpose. Data collected include information on the type of abortion care, abortion technology, age category, gestational age of the pregnancy, and postabortion contraceptive method chosen by each woman.

### Data Management, Analysis, and Use

Data were entered into a Microsoft Excel database and a national online database to conduct descriptive analysis, review trends, and monitor progress. Service data were analyzed and used at facility, district, zonal, and regional levels to review achievements and identify challenges requiring action. Wall charts were developed and displayed at the facilities to track service utilization and postabortion contraceptive methods chosen using data in the aggregate. Regional program review meetings for CAC/PAFP providers and zonal and district program staff took place semiannually.

PAFP uptake and indicators of the pattern of method use presented in this article are based on descriptive analysis of these service data. The study team focused on changes in the distribution of method use in the health centers compared with the hospitals, as well as PAFP uptake compared with results of the stand-alone family planning program in the same facilities. Bivariate analysis used chi-square tests to examine the differences in method use patterns between different categories. Statistical significance was established at *P*<.05.

Data were collected by the program for the purpose of continuous quality improvement, not for the purpose of conducting systematic research in a strictly defined model. All data analysis was conducted according to international principles of maintaining privacy and confidentiality of personal information.

## RESULTS

Between July 2009 and June 2014, a total of 44,682 women sought abortion care in 101 public health facilities in the region. Among all women who sought care, 34,212 (77%) received safe and legal induced abortions; the remaining 10,470 women (23%) received postabortion care for complications of unsafe abortions or complicated miscarriages. Most women, 60%, were young; only 40% of women who sought abortion care during this period were older than 24.

### Uptake Increased and Method Mix Improved

**FIGURE 1.**
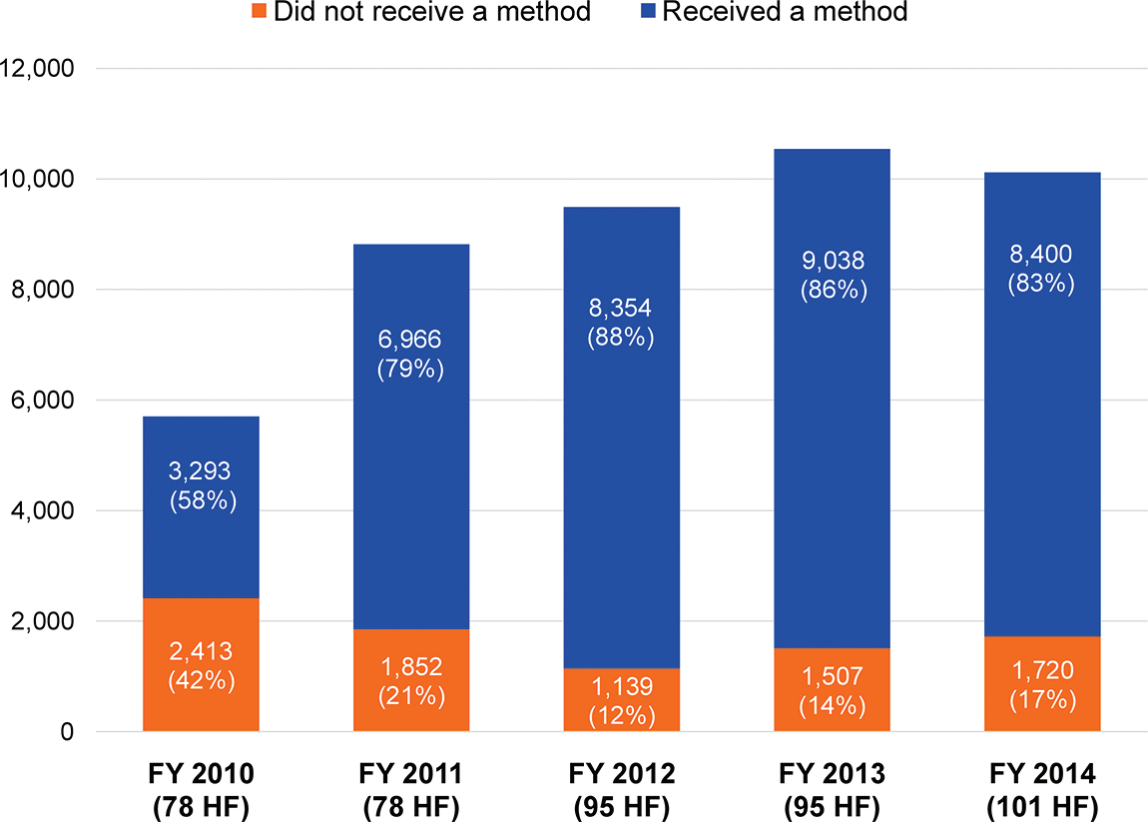
Proportion of Comprehensive Abortion Care Clients Who Left the Facility With a Contraceptive Method by Year, SNNPR, Ethiopia, FY 2010–14 (N=44,682) Abbreviations: FY, fiscal year; HF, health facilities; SNNPR, Southern Nations, Nationalities, and People’s Region.

Service delivery data showed progressive improvement in the uptake of postabortion contraception in intervention facilities. The proportion of abortion clients who left the intervention facilities with some form of contraception increased from 58% in FY 2010 to 83% in FY 2014 (Figure 1).

The proportion of clients who left with contraception increased, and the method mix improved.

**FIGURE 2.**
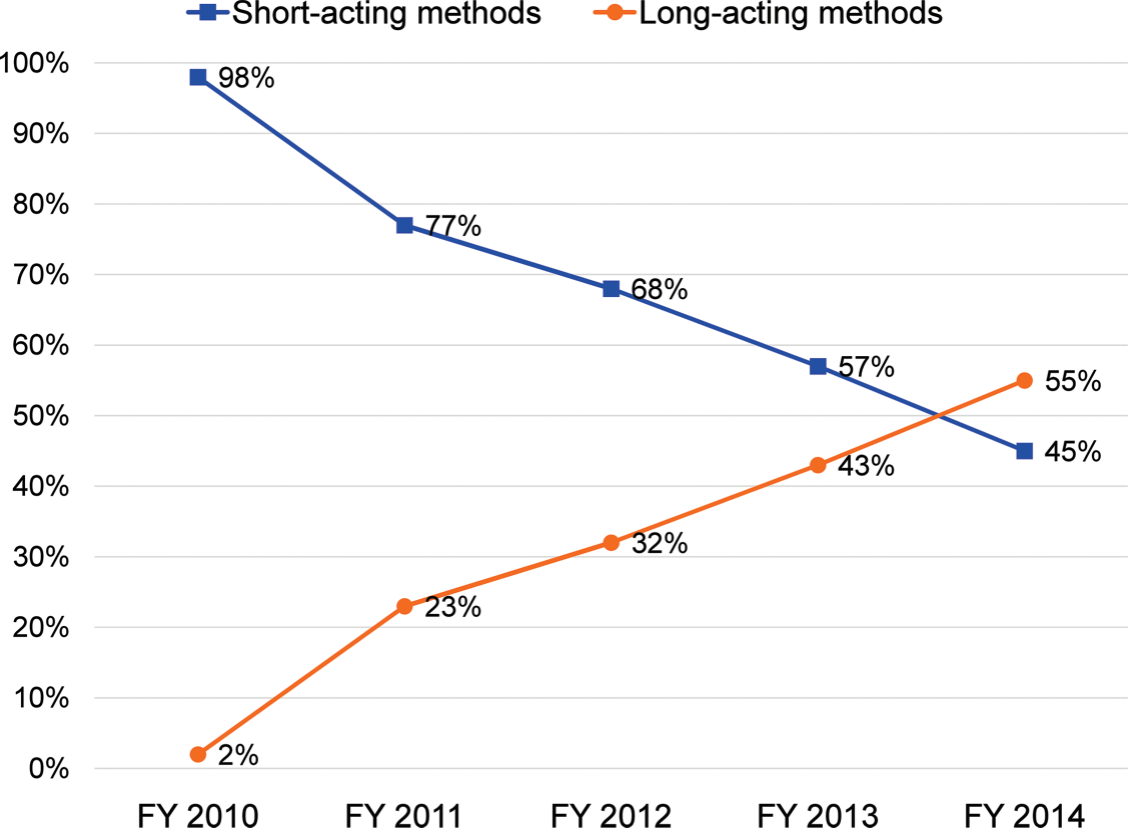
Trends in Use of Short‐Acting and Long‐Acting Methods Among Postabortion Contraceptive Acceptors by Year, SNNPR, Ethiopia, FY 2010–14 (N=36,051) Abbreviations: FY, fiscal year; SNNPR, Southern Nations, Nationalities, and People’s Region.

The package of interventions also progressively changed the contraceptive method mix, particularly the proportion of women accepting LARC methods. Figure 2 shows that the proportion of CAC clients who accepted a method and chose short-acting hormonal methods, mainly injectable and oral contraceptives, declined from 98% in FY 2010 to 45% in FY 2014. The proportion of all CAC contraceptive acceptors who chose a long-acting method rose from 2% in FY 2010 to 55% in FY 2014 (*P*<.001). The average annual rate of increase in LARC method acceptance was 13.3%; the highest increment, of 21%, occurred between FY 2010 and FY 2011. In addition, among all CAC clients, in FY 2010 about 57% chose short-acting methods, while only 1% chose LARCs. After 5 years of the intervention, the proportion choosing short-acting methods decreased to 37%, whereas the percentage choosing LARCs climbed to 46%.

**FIGURE 3.**
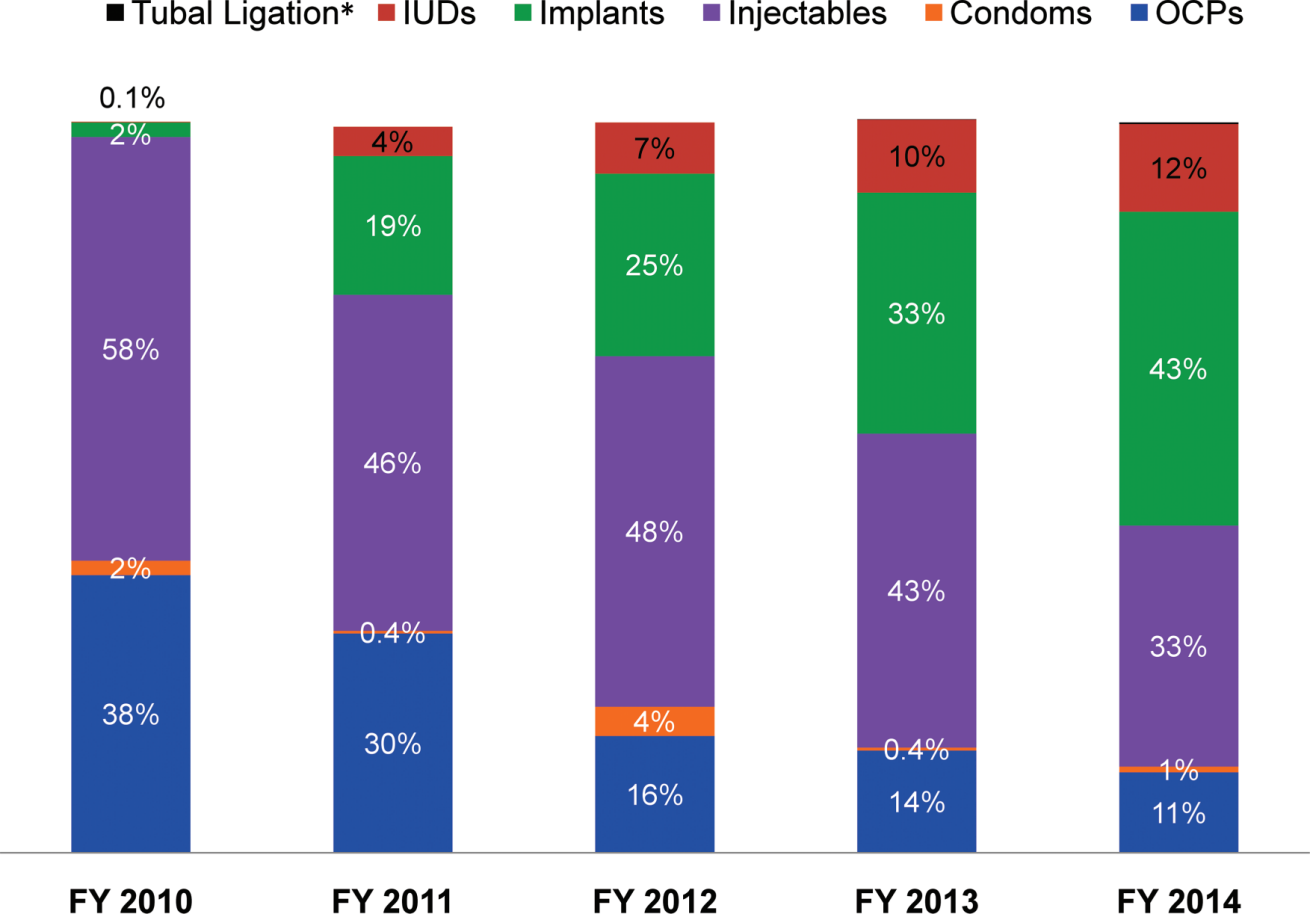
Trends in the Method Mix Among Postabortion Contraceptive Acceptors by Year, SNNPR, Ethiopia, FY 2010–14 (N=36,051) Abbreviations: FY, fiscal year; IUDs, intrauterine devices; OCPs, oral contraceptive pills; SNNPR, Southern Nations, Nationalities, and People’s Region. * Values for tubal ligation were 0% in 2010, 2011, and 2012; 0.03% in 2013; and 0.2% in 2014.

Figure 3 illustrates the changes in the method mix specifically for long-acting and permanent methods during the intervention period. The share of implants as a percentage of all method use rose quickly, from 2% in FY 2010 to 43% in FY 2014, while IUD acceptance increased from only 0.1% in FY 2010 to 12% in FY 2014. Female sterilization (tubal ligation) remained the least chosen method, with very few CAC clients (3 in FY 2013 and 17 in FY 2014) choosing voluntary surgical contraception to limit childbearing.

### Contraceptive Acceptance Higher in Health Centers Than in Hospitals

**FIGURE 4.**
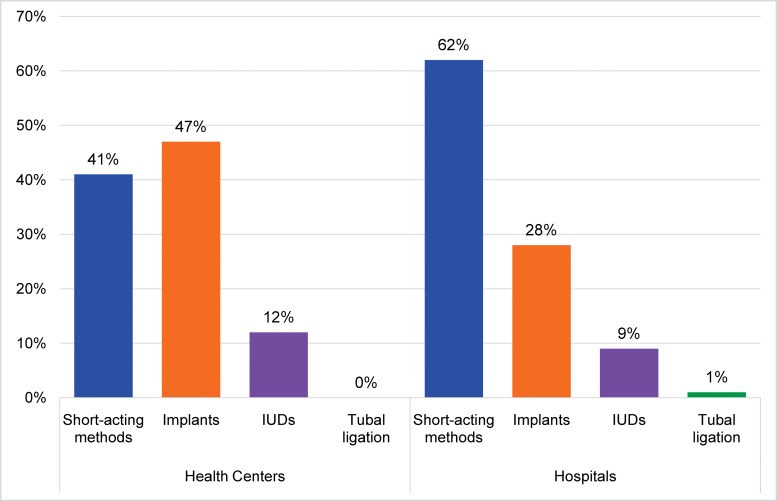
Contraceptive Method Use Patterns Among Postabortion Contraceptive Acceptors by Type of Facility, SNNPR, Ethiopia, FY 2014 Abbreviations: IUDs, intrauterine devices; SNNPR, Southern Nations, Nationalities, and People’s Region.

Disaggregating postabortion contraceptive service data by facility type revealed higher contraceptive acceptance in health centers (86% of all women left with a method) than in hospitals, where 71% of abortion clients left with contraception. Figure 4 shows that the method mix was more balanced in health centers than in hospitals. For example, in FY 2014 the proportion in hospitals choosing long-acting methods was only 37%, while 59% of women cared for in health centers chose a LARC method after their abortion care (*P*<.01).

### Choice of LARC Methods Higher in Comprehensive Abortion Care Areas Than in Routine Services

**FIGURE 5.**
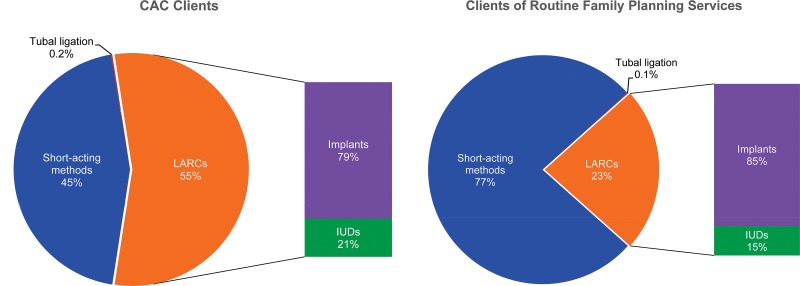
Contraceptive Method Use Patterns Among Comprehensive Abortion Care Clients Versus Clients of Routine Family Planning Services, SNNPR, Ethiopia, FY 2014 Abbreviations: IUDs, intrauterine devices; LARCs, long‐acting reversible contraceptives; SNNPR, Southern Nations, Nationalities, and People’s Region.

As Figure 5 shows, comparing LARC acceptance in the comprehensive abortion care areas with the routine family planning services in the same sites indicates that the rate of LARC acceptance among postabortion clients, averaging 55%, was much higher than in the same facilities’ routine family planning programs, where on average only 23% chose LARC methods (*P*<.001). Among those choosing long-acting methods, the proportion choosing IUDs (21%) was also higher among postabortion clients than in routine family planning services, where only 15% of such clients chose IUDs. Trend analyses of LARC uptake in the routine programs showed that the proportion of implant acceptors increased from 3% in FY 2010 to 19% in FY 2014, while IUD acceptance increased far more slowly, from only 0.1% to 4% of all methods over the same time period.

## DISCUSSION

The package of interventions introduced in service expansion in SNNPR during the past half-decade tested the impact of focused attention to postabortion service quality on the uptake of contraceptive methods, particularly on the choice of long-acting methods. In addition to increased attention to postabortion contraception, the rights-based approach to informed consent and decision making emphasized the importance of contraceptive choice and ensured providers’ commitment to the sexual and reproductive rights of women. While the purpose of the project was to improve postabortion service quality overall, benefits included an increase in postabortion choice of a contraceptive method and a greater proportion choosing the more effective, long-acting methods.

As the result of this intervention, the proportion of abortion clients who received contraception increased from 58% to 83%, a rate in line with other countries’ achievements implementing similarly intensive strategies to improve services.^11^^,^^12^^,^^14^^,^^16^ For instance, in a similar pilot initiative in Turkey to integrate family planning with abortion services, the proportion of abortion clients who accepted contraceptive methods rose from 65% in 1991 to 98% in 1992.^11^ Findings from around the world show that provision of family planning counseling and services immediately after all types of abortion care can increase the postabortion contraceptive acceptance rate from 0%–10% pre-intervention to 50%–80% within 1 to 2 years after the intervention.^7^^,^^10^

Providing family planning counseling and services immediately after abortion care can increase the postabortion contraceptive acceptance rate from 0%–10% pre-intervention to 50%–80% within 1–2 years.

### Increased Choice of Long-Acting Methods

One notable achievement of this particular program intervention was the significant uptake of long-acting methods, which were greatly underutilized before the intervention. Over the intervention period, some CAC clients who would have chosen short-acting methods now chose LARCs instead. As the overall proportion of PAFP acceptors increased, some new CAC clients who would not have taken any method now also chose LARCs. Key strategies to this end were improving the availability of long-acting methods and related equipment, providing clinical and contraceptive training for mid-level providers, and encouraging and facilitating provision of all contraceptive methods in the same location where abortion services are provided. Similar components of the intervention in Turkey significantly improved the acceptance of LARC methods.^11^ Furthermore, a randomized trial in China found that a comprehensive postabortion care package increased the use of more effective contraception, such as IUDs and implants, when compared with a minimum package of services.^19^

LARC methods should be an integral part of a comprehensive method mix since they are more effective than short-acting methods and so better prevent repeat abortions. A study in the clinics of Planned Parenthood Northern California found that immediate insertion of IUDs postabortion significantly reduced unwanted pregnancy compared with other contraceptive methods or delayed IUD insertion; women who received an IUD immediately postabortion had only 35 abortions per 1,000 woman-years of follow-up compared with 91 for the comparison group.^22^

Women who sought postabortion care at health centers in SNNPR were more likely to accept postabortion contraception and to choose a long-acting method than women who sought postabortion care in hospitals. While this finding needs further investigation, there is evidence in other parts of Africa as well that decentralization of services from hospitals to lower-level facilities has increased the uptake of postabortion contraception. For example, a pilot project in Tanzania to assess the feasibility of expanding PAC services to health centers and dispensaries reported that 91% to 100% of clients received postabortion counseling, and 97% to 100% of those clients left the locations with a method, except in the few facilities that did not adhere to the intervention.^31^ Moreover, public health experts agree that abortion care and contraceptive services need to be decentralized to improve availability and quality by making services more accessible to women in rural areas and to reduce overcrowding in tertiary hospitals.^6^

Most women who chose a long-acting method chose an implant (79% of all LARC acceptors). Women’s method preferences were likely to be affected not only by the counseling they received but also by educational information campaigns in the region.^15^ During the study period, the federal and regional governments conducted extensive educational campaigns on contraceptive implants to promote use of this method. In fact, a recent study exploring knowledge and acceptance of long-acting and permanent methods in southeastern Ethiopia found that reproductive-age women were most knowledgeable about contraceptive implants. Some 87% of married women reported that they had heard of Norplant contraceptive implants.^32^

In the project sites, IUDs have become a more common selection and are now the choice of 21% of all LARC method acceptors in the abortion care areas of these facilities. However, lack of awareness and local misconceptions about the IUD, such as fear of infertility and infection, still persist across Ethiopia.^32^

Similar service improvement strategies have been employed to shift the method mix and increase use of long-acting methods in routine family planning programs in the region. The achievement has not been as satisfactory as in abortion care areas, however. It seems likely that the longer interactions between providers and abortion clients in the abortion care settings results in more thorough counseling; clients are counseled during the pre-, intra- and post-procedure times. This finding is in line with those of a study in southeastern Ethiopia that selection of long-acting and permanent methods was significantly associated with the number of times that clients and providers discussed these methods.^32^ Also, abortion clients may have a higher unmet need for long-acting methods because of their youth; a large proportion are unmarried but sexually active, wanting to delay marriage and childbearing.

### Limitations of the Study

The findings presented here provide results from a large intervention for women who sought either induced abortion or postabortion care in more than 100 public health facilities. These findings are based on service data from intervention sites; they may not be generalizable to all facilities in the region, the country, or the private sector. However, this research was conducted in partnership with the Ethiopian MOH at all levels, and, as such, it should be seen as describing a replicable, low-cost model for scaling up services in Ethiopia and elsewhere. No efforts were made to control for community or national efforts to increase family planning acceptance in the general population, and so it is not possible to attribute all of the gains made in this study period to the package of service delivery interventions. Findings are meant to describe and provide a platform for comparison and lessons learned. The large size of the program and the scope of data collection make possible further research to explore the association of sociodemographic factors (such as marital status, parity, education, occupation, previous use of contraception, and abortion history) with the type of abortion care, procedure, and contraceptive method acceptance. This program has recently begun a phase-out strategy aimed at ensuring self-reliance among high-performing facilities to sustain delivery of comprehensive abortion care without routine NGO support.

This research was conducted in partnership with the Ethiopian MOH at all levels and, as such, should be seen as describing a replicable, low-cost model for scaling up services in Ethiopia and elsewhere.

## RECOMMENDATIONS AND CONCLUSIONS

### Family Planning Programs Can Fail in 3 Ways

Reproductive health organizations and experts have emphasized 2 potential failures in any family planning program that allow the vicious cycle of unintended pregnancy and abortion to persist. The first failure would be the **inability of the program to prevent unwanted or mistimed pregnancy.** The second potential failure would be **when a woman who received abortion services leaves without the means to prevent another pregnancy** in the future.^33^ However, there is also a third potential failure, which may result in lower levels of satisfaction and acceptance and, thus, in further unwanted pregnancies: **when a program cannot provide a wide range of contraceptive methods for postabortion clients**.

Operations research conducted in postabortion settings has shown that offering a wide range of methods coupled with effective counseling increases contraceptive uptake.^12^^,^^16^ A limited choice of methods can lead to a woman selecting a method that she may be more likely to discontinue because it is ultimately less acceptable to her or her partner. Additionally, offering only short-acting methods to women who need and are eligible for long-term protection may result in unwanted pregnancy because the return to fertility after abortion is immediate and the chance of method failure is high with short-acting methods. To avoid this pitfall, it is vital to establish effective programs that offer a wide range of methods.

The quality of comprehensive abortion care may be limited by infrastructure that is often outdated and inappropriate for services that have moved from labor and delivery or surgical areas to stand-alone, predominately outpatient service areas. Comprehensive and acceptable abortion care requires that adequate infrastructure, a wide range of contraceptive methods in the procedure area, and all necessary equipment be available consistently. Providers well trained in both abortion and contraceptive services and who are motivated and supported to provide services are also key to success.

Comprehensive abortion care requires a wide range of contraceptive methods, adequate infrastructure, and well-trained providers, among other factors.

### Research Needed on Postabortion Method Mix

To achieve positive results, postabortion services need to promote a contraceptive method mix acceptable and effective for young and single women who need long-term and reliable protection for their long reproductive lives ahead. LARC methods are private and provide continuous protection against unwanted pregnancy, with higher continuation rates than short-acting methods, while serving a range of clients’ intentions to delay or space births.

Young and single women need long-term and reliable protection for their long reproductive lives ahead.

However, evidence on the most acceptable method mix for postabortion clients is scarce. More health systems research is needed to assess method continuation and discontinuation rates and to evaluate the impact of improvements in PAFP services on contraceptive prevalence and whether such improvements result in lower maternal morbidity and mortality.^34^

**Figure:**
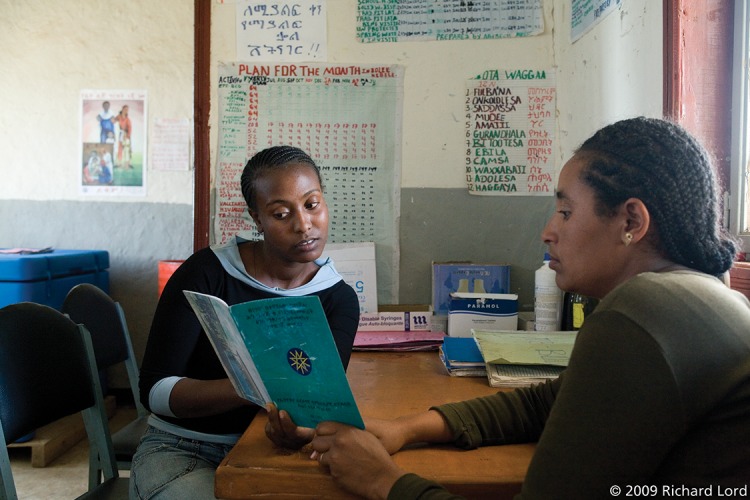
A mid-level provider discusses available contraceptive methods with a client to assist her in making an informed, voluntary contraceptive choice.

### Balanced Method Availability Helps Prevent Repeat Abortions

The package of interventions described here has set the groundwork for stronger postabortion contraception services. Postabortion contraception contributes to the prevention of unwanted pregnancy. Also, it contributes to increased contraceptive acceptance on a national level by addressing an often overlooked group of women with high demand for contraception. Focusing on promoting a more balanced method mix and improving access to LARC methods has led to higher levels of postabortion contraceptive method acceptance overall.

In conclusion, well-planned and organized PAFP services are a cost-effective, feasible, and easily replicable strategy for serving women, especially young women, with an unmet need for contraception who are highly vulnerable to a future unintended pregnancy and repeat abortion.

## Notes

**Peer Reviewed**
